# Accumulation of retrotransposons contributes to W chromosome differentiation in the willow beauty *Peribatodes* *rhomboidaria* (Lepidoptera: Geometridae)

**DOI:** 10.1038/s41598-023-27757-3

**Published:** 2023-01-11

**Authors:** Martina Hejníčková, Martina Dalíková, Magda Zrzavá, František Marec, Pedro Lorite, Eugenia E. Montiel

**Affiliations:** 1 Faculty of Science, University of South Bohemia, České Budějovice, Czech Republic; 2 Institute of Entomology, Biology Centre CAS, České Budějovice, Czech Republic; 3 Department of Experimental Biology, Genetics Area, University of Jaén, Jaén, Spain

**Keywords:** Molecular evolution, Evolutionary genetics, Chromosomes, Cytogenetics, Genome

## Abstract

The W chromosome of Lepidoptera is typically gene-poor, repeat-rich and composed of heterochromatin. Pioneering studies investigating this chromosome reported an abundance of mobile elements. However, the actual composition of the W chromosome varies greatly between species, as repeatedly demonstrated by comparative genomic hybridization (CGH) or fluorescence in situ hybridization (FISH). Here we present an analysis of repeats on the W chromosome in the willow beauty, *Peribatodes* *rhomboidaria* (Geometridae), a species in which CGH predicted an abundance of W-enriched or W-specific sequences. Indeed, comparative analysis of male and female genomes using RepeatExplorer identified ten putative W chromosome-enriched repeats, most of which are LTR or LINE mobile elements. We analysed the two most abundant: PRW LINE-like and PRW Bel-Pao. The results of FISH mapping and bioinformatic analysis confirmed their enrichment on the W chromosome, supporting the hypothesis that mobile elements are the driving force of W chromosome differentiation in Lepidoptera. As the W chromosome is highly underrepresented in chromosome-level genome assemblies of Lepidoptera, this recently introduced approach, combining bioinformatic comparative genome analysis with molecular cytogenetics, provides an elegant tool for studying this elusive and rapidly evolving part of the genome.

## Introduction

Sex chromosomes have evolved independently in multiple taxa^1^. In most cases, their evolution seems to follow similar trajectories with predictable outcomes, although various exceptions and derivations are relatively common^2^. Nevertheless, the classical model of sex chromosome evolution assumes that the sex-determining factor is acquired by one of the homologous chromosomes, resulting in a new pair of sex chromosomes^3^. The new heteromorphic chromosome (usually Y or W, depending on the type of heterogamety) eventually begins to attract sexually antagonistic genes, and these evolutionarily advantageous mutations can be further fixed, e.g. by inversions. However, such progressive differentiation simultaneously restricts recombination in this region. This is usually followed by gradual degenerative changes such as the accumulation of repetitive sequences and pseudogenization, often leading to heterochromatinization and subsequently even to the dispensability of this particular chromosome. If the sex-determining factor is translocated or substituted, such a sex chromosome may eventually be lost forever and the entire sex chromosome life cycle may be restarted^2,4^.

The processes of sex chromosome evolution are best studied in diverse groups of organisms that likely share an ancestral karyotype while exhibiting various abnormalities at different evolutionary stages. One of these is the insect order Lepidoptera (moths and butterflies), the largest group with female heterogamety^5^. Until recently, however, very little was known about the actual molecular composition of their W chromosomes, which were regularly excluded from NGS projects due to their repetitive nature causing assembly problems. So far, only a few genes have been found on the W chromosome, such as the sex-determining female factor generating *Fem* piRNA^6^. In contrast, the lepidopteran W chromosomes are typically filled with various types of ubiquitous transposable elements (TE)^7–9^ and may also contain satellite DNA^10^. Occasionally, some of these sequences are enriched on the W chromosome, such as piSAT1 in *Plodia* *interpunctella*^11^, or even W-specific (i.e. present only on the W), such as CpW2 and CpW5 in *Cydia* *pomonella*^12^.

Apart from Lepidoptera, the accumulation of transposable elements and other repetitive DNA by the heterogametic sex chromosomes has been observed in plants^13^, fungi^14^, humans^15^, other mammals^16^, birds^17^, *Drosophila* flies^18^, beetles^19^ and many others^20^. Such events usually lead to the formation of heterochromatin, which may attract even more TEs, as some of them preferentially insert into silenced regions^21^. This could be the result of opposing selection forces balancing the deleterious effects of integration on the host cell on the one hand, and the propagation of the specific transposable element in the genome on the other. Some degree of preference for integration sites can be observed in almost all TE types^22^. Their population dynamics within the genome are described by the deleterious insertion model, which assumes that transposons are eliminated from gene-rich regions by selection pressure, or by the ectopic recombination model, which assumes a higher abundance of transposons in regions with low recombination rates where they cannot be removed by ectopic recombination^23^. These models offer an explanation for the accumulation of transposable elements on heterogametic sex chromosomes (i.e. Y and W).

Hence, transposable elements may play an important role in the evolution and differentiation of sex chromosomes. In fact, it has already been shown that TEs can influence whole genomes, both in destructive and constructive ways. Destructive forces primarily include insertions into genes or their promoters that impede their function, which may eventually lead to lower fitness or the development of various diseases^24^. On the other hand, some of our vital proteins originated via domestication of ancient transposable elements, such as RAG proteins that carry out recombination in immunoglobulin genes^25^, or telomerase which maintains chromosome ends^26^. In addition, they may contribute to genome size expansion or speciation, as e.g. LINE-1 retrotransposons may reduce gene expression, provide new exons for protein-coding genes^27^, or serve as “booster stations” for the spread of silencing Xist RNA during X chromosome inactivation in mammals^28^. To sum up, transposable elements can shape genomes in various ways and influence sex chromosome differentiation, potentially leading to speciation or sexual dimorphism^29^.

Previous cytogenetic studies in Lepidoptera have shown striking differences between W chromosome composition and size even between closely related species, suggesting rapid evolution^30–32^. One of the most informative cytogenetic tools is comparative genomic hybridization (CGH), which enables to estimate the differentiation level and the ratio of common to female-enriched sequences on the W chromosome. In our recent study^30^, CGH revealed a high degree of sex chromosome differentiation in many species of Geometridae (Lepidoptera), including the willow beauty (*Peribatodes* *rhomboidaria)*. This species has a standard lepidopteran karyotype (n = 31, WZ/ZZ), and its normal-sized W chromosome is strongly DAPI positive and contains a large amount of heterochromatin. Furthermore, it shows a remarkable hybridization pattern after CGH, as it is strongly marked by the female genomic probe (Fig. S1; Fig. 8e in our recent study^30^), suggesting the presence of female-enriched and/or female-specific sequences. The question remained open as to which type of repetitive sequences mainly contribute to the molecular differentiation of the W chromosome in *P.* *rhomboidaria*. To address this issue, we performed low coverage NGS sequencing of three individuals of each sex and compared their repeat content using the RepeatExplorer pipeline^33^. The results describe the repeatome of *P.* *rhomboidaria* in general and reveal two W-enriched sequences, contributing to our knowledge of W chromosome evolution in Lepidoptera. Finally, this study validates a novel approach for analysing differences in repeat content that can be applied to other species.

## Materials and methods

### Insects

Adult specimens of *P.* *rhomboidaria* were collected in the field from June to September 2019–2020 in the vicinity of České Budějovice, Czech Republic, using light traps and entomological nets. Females were kept in plastic containers with host plants (mostly leaves of *Achillea* *millefolium*) until they laid eggs. After hatching, the larvae were reared to the penultimate instar stage, dissected for chromosome preparations and the remaining tissues were frozen in liquid nitrogen and stored at − 20 °C.

### Chromosome preparations

Meiotic chromosomes were obtained from gonads of penultimate instar larvae and spread chromosome preparations were made as previously described^34^, dehydrated in an ethanol series (70%, 80% and 100%, 30 s each), air-dried and stored at − 20 °C until further use.

### DNA isolation and sequencing

Genomic DNA (gDNA) was extracted from one half of the larva using CTAB (hexadecyltrimethylammonium bromide; Sigma-Aldrich, St. Louis, MO, USA) according to the published protocol^35^ with modifications previously described^30^. The concentration of isolated DNA was measured using Qubit 3.0 fluorometer (Invitrogen, Carlsbad, CA, USA), and its quality was assessed by the absorbance ratio at 260/280 nm using a Nanodrop 2000 spectrophotometer (Thermo Fisher Scientific, Waltham, MA, USA). For each of the three broods studied (i.e. offspring of the same mother), we selected a sample of optimal quality from one male and one female (6 samples in total). Paired-end sequencing of 150 bp long reads from a library with 450 bp inserts was performed by Novogene (HK) Co., Ltd. (Hong Kong, China) using the Illumina HiSeq 4000 platform.

### NGS data processing and repeatome analysis

The obtained Illumina raw reads were first processed by Trimmomatic version 0.32^36^, the Illumina sequencing adapters were trimmed and finally all reads were cropped to a final length of 140 bp. The quality of the reads before and after processing was checked using FastQC version 0.10.1^37^. The remaining paired sequences were converted to FASTA format using FastQtoFasta software from the FASTX-Toolkit version 0.0.14 (http://hannonlab.cshl.edu/fastx_toolkit). The following procedures were performed using the RepeatExplorer2 Utilities Kit version 0.3.8-451 (https://repeatexplorer-elixir.cerit-sc.cz). Sequences were interlaced and a random sample of 100,000 reads was generated for each individual (corresponding to approximately 0.02 × genome coverage) and tagged with a specific read name prefix. Alternatively, read sampling was done using the seqtk tool (https://github.com/lh3/seqtk). Samples were concatenated into a single fasta file, which was processed by RepeatExplorer^33^ using the default parameters for comparative analysis. To identify W-enriched repeats, the number of reads in individual clusters was compared between males and females using R version 4.0.3 in RStudio version 1.4.1103 (https://www.rstudio.com). Only clusters that consisted of at least 0.01% of the analysed reads and showed statistically significant differences (*P* < 0.05,two sample *t*-test with unequal variance) were considered. The annotation of the repeats was based on the automatic annotation of RepeatExplorer using Metazoa v3 database. Annotation of selected repeats was further confirmed by protein-based repeat masking in RepeatMasker 4.1.2^38^ (https://www.repeatmasker.org).

### PCR and cloning

Based on the RepeatExplorer results, we further analysed the two most abundant W-enriched repeats: cluster 32 (containing the PRW LINE-like retrotransposon) and cluster 79 (containing the PRW Bel-Pao retrotransposon). We designed primers (Table S1) for a selected contigs from cluster 32 and the assembly of three contigs covering most of the cluster 79 using Geneious Prime version 2021.1.1. (https://www.geneious.com). These primers were used in a standard 25 μl PCR mix containing 1× Ex*Taq* buffer (TaKaRa, Otsu, Japan), 2U DNA Ex*Taq* Polymerase (TaKaRa), 200 μM of each nucleotide, 1 μM of each primer and 50 ng of gDNA with the following profile: initial denaturation at 94 °C for 3 min, followed by 30 cycles of 94 °C for 30 s, 56 °C for 30 s and 72 °C for 1 min 30 s; final extension was at 72 °C for 3 min. The results were checked on a standard 1.5% agarose gel in 1× TAE buffer. Gels were stained with ethidium bromide for 20 min and photographed under UV light. To avoid cloning problems due to the size of the PRW Bel-Pao sequence*,* we generated two shorter overlapping PCR products (using primer pairs PRW_F1 + PRW_R1 and PRW_F2 + PRW_R2; Table S1). All products were purified using ExoSAP-IT (ThermoFisher Scientific) and cloned into a vector using pGEM-T Easy Vector System (Promega, Madison, WI, USA) according to the manufacturer's instructions. Plasmid DNA was isolated using the NucleoSpin Plasmid kit (Macherey–Nagel, Düren, Germany) according to the manufacturer's protocol. The identity of the fragment was verified by Sanger sequencing in SEQme (Dobříš, Czech Republic) with universal M13 primers. All examined sequences (both contigs and cloned fragments) were deposited in GenBank (Table 1).

**Table 1:** Accession numbers of studied sequences.

Name	Origin	Accession number
PRW LINE-like	Clone	OP361285
PRW Bel-Pao part I	Clone 1	OP361282
PRW Bel-Pao part II	Clone 2	ON815295.1
PRW Bel-Pao part I with deletion	Clone 3	OP361284
PRW Bel-Pao consensus	Assembly of clone 1 and 2	OP361283
PRW Bel-Pao consensus with deletion	Assembly of clone 3 and 2	OP410344
PRW LINE-like contig from RE	In silico RepeatExplorer from cluster 32 contig	OP410342
PRW Bel-Pao contig consensus from RE	Assembly of 3 in silico RepeatExplorer contigs from cluster 79	OP410343

### Coverage analysis

To obtain more information about the representation of selected fragments in the genomes of both sexes, we mapped the trimmed, filtered and paired reads (see above) to the cloned consensus sequences in Geneious Prime version 2021.1.1. using the built-in algorithm. Mapping was done separately for male and female reads (three combined samples from each sex) using the 95% identity threshold, and the mapped reads were manually sorted to ensure accurate results. We also estimated copy number based on total coverage.

### Probe labelling and fluorescence in situ hybridization (FISH)

PRW LINE-like mapping was done in Jaén, Spain. 1 µg of PRW LINE-like DNA was labelled by nick translation using the biotin-NT-mix (Roche, Basel, Switzerland). The probe was then precipitated along with 50 μg yeast RNA and 50 μg salmon sperm DNA and redissolved in 50% formamide in 2 × SSC. Fluorescence in situ hybridization (FISH) was performed according to the published protocol^39^ with listed modifications^40^.

PRW Bel-Pao mapping was done in České Budějovice, Czech Republic. Both fragments (I and II, which do not contain the deleted region—see below) were labelled by PCR containing 0.04 mM each of dATP, dCTP and dGTP; 0.014 mM dTTP, 0.025 mM biotin-16-dUTP (Jena Bioscience, Jena, Germany), 1× Ex*Taq* buffer (TaKaRa), 1–10 ng plasmid DNA, 1 μM of each primer and 2U DNA Ex*Taq* Polymerase (TaKaRa) under the same conditions as described above. FISH was performed according to the published protocol^41^, with signal amplification using Cy3-conjugated streptavidin (Jackson ImmunoRes. Labs. Inc, West Grove, PA, USA) at a dilution of 1:1000 with washing blocking buffer.

## Results

### Characterization of repeats in the genome of *P. rhomboidaria*

Total genome coverage after read processing was 2.38× for females and 2.24× for males, corresponding to approximately 0.77 × coverage depth/per sample, based on a rough estimate of genome size 1C ~ 0.646 pg performed by flow cytometry in the laboratory of Petr Koutecký according to the published protocol^34^.

The RepeatExplorer analysis clustered about 44% of input reads, indicating the relative representation of repeats in the genome.

Of all the identified clusters, 220 (representing ~ 24% of the input reads) were subjected to automatic annotation and further analysis, as each of these clusters represented at least 0.01% of the examined reads. However, the automatic annotation was not able to classify most of the top 220 clusters and clusters corresponding to 87% of the input reads remained unknown. Among the annotated clusters, about 0.91% of the input reads were satellites. As for the classified mobile elements, the prevailing groups were long interspersed nuclear elements (LINE) with 1.33% of the reads and long terminal repeats (LTR) with 0.65% of the reads.

### Characterization of selected sequences

**Figure 1:**
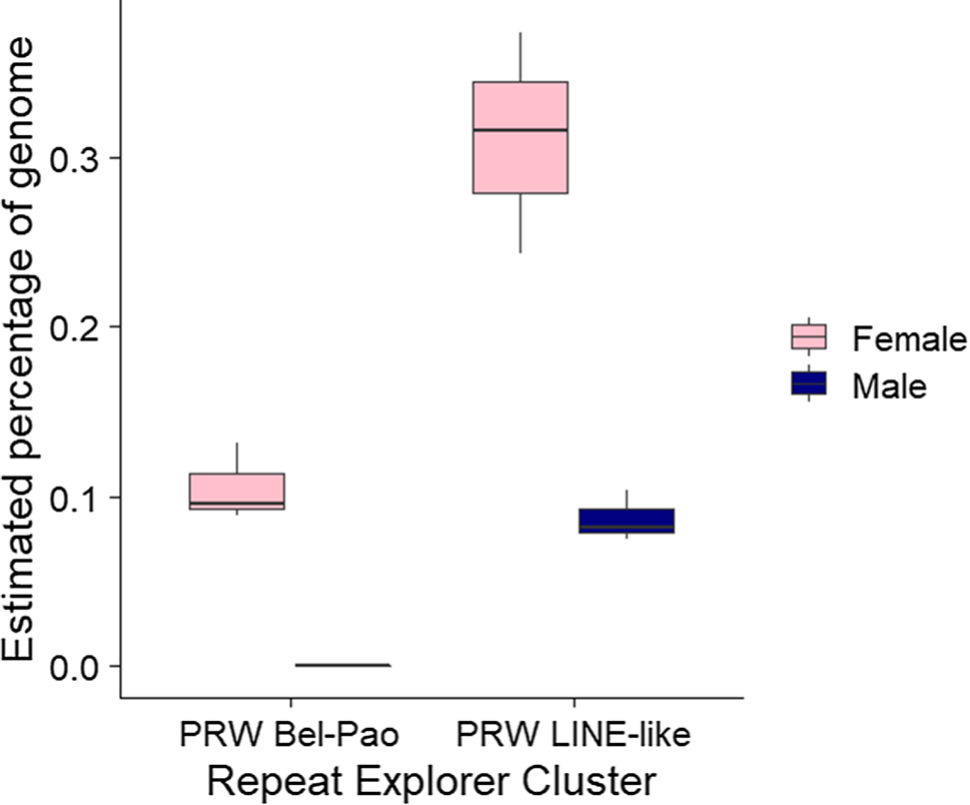
Boxplot of estimated genome percentages of the two most abundant putative W repeats based on RepeatExplorer results from 3 females and 3 males of *Peribatodes* *rhomboidaria*. Median, interquartile range, and smallest and largest values in 1.5 × interquartile range are shown.

Out of the top 220 clusters, 12 clusters were statistically significantly female enriched and three clusters were male enriched (Fig. S2). Since the maximum male enrichment was 1.37× , any female-enriched cluster where the female-to-male ratio was below this value was considered a false positive. In the end, we obtained ten clusters representing putative W repeats (Fig. S2). The average female enrichment in these clusters was 6.48× and the maximum enrichment was 197.21×. Most of the putative W repeats were annotated as retrotransposons (Table 2). The two most abundant W repeats represented by cluster 32 (PRW LINE-like) and cluster 79 (PRW Bel-Pao) were further analysed (Fig. 1).

**Table 2:** Putative W chromosome enriched repeat clusters.

Cluster no	Female enrichment	Female genome (%)	Male genome (%)	Annotation
**32**	**3.57× **	**0.31**	**0.09**	**LINE and/or DNA/Transib**
**79**	**197.21× **	**0.11**	**0.001**	**LTR/Bel-Pao**
93	5.66×	0.07	0.01	LTR/Ty3-Gypsy
97	1.84×	0.05	0.03	LTR/Ty3-Gypsy
127	3.91×	0.04	0.01	LTR/Ty3-Gypsy
161	58.43×	0.03	0.001	Unknown
178	13.13×	0.02	0.002	LTR/Ty3-Gypsy
182	1.94×	0.02	0.009	Unknown
199	1.9×	0.01	0.008	LTR/Bel-Pao
207	1.6×	0.01	0.008	LINE

Cluster numbers, their enrichment in the female genome compared to the male genome, their representation in the female and male genomes in % and their annotation according to RepeatMasker. Further examined clusters in bold.

Based on the RepeatExplorer results, cluster 32 (corresponding to PRW LINE-like) was enriched 3.57× in the female data, where it represented about 0.31% of the genome, while in males it was only about 0.09% (Table 2, Fig. 1). Since the RepeatExplorer results are based on data with very low coverage, we mapped all processed reads to the cloned consensus sequence to obtain more accurate information on the abundance of this repeat. The results showed significantly higher coverage in females (774.6×) than in males (39.2×). We also estimated copy number using total coverage information, which was 325 copies in females and 17 copies in males, making up to a 19-fold difference.

**Figure 2:**
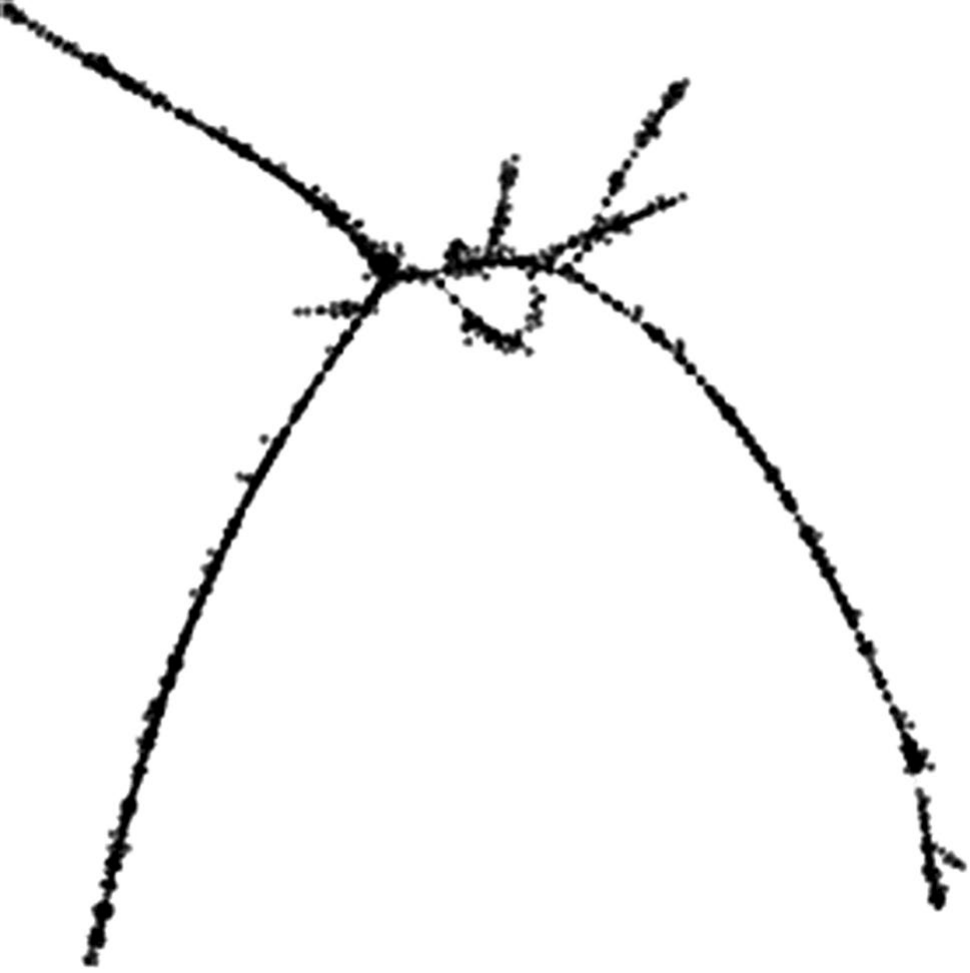
PRW LINE-like RepeatExplorer graph. Graph layout for cluster 32 generated by RepeatExplorer. Nodes represent individual illumina reads and edges their sequence overlap.

Automatic annotation by RepeatExplorer classified the cluster 32 repeat as a retrotransposon from the LINE element group, based on the homology of the two identified reverse transcriptase (RT) domains. However, the structure of the RE graph was relatively complicated (Fig. 2), similarly to the RepeatMasker-based annotation. Apart from the expected two LINE RTs from the LINE/CR1 (which corresponds to our cloned and FISH-mapped sequence) and LINE/L2 subgroups, RepeatMasker also found a DNA transposon similar to Transib in this cluster.

Database search in NCBI BLASTN^42^ revealed 72% identity with a non-LTR retrotransposon T1Q-like reverse transcriptase gene of *Abraxas* *sylvata* (GenBank Acc. No. HQ284333.1), another moth from the family Geometridae. All other relevant hits without closer annotation came from the Tree of Life project^43^ and ranged from 99% sequence identity in *P.* *rhomboidaria* to 70% identity in many related species.

Cluster 79 (corresponding to PRW Bel-Pao) was the most female-enriched repeat cluster identified by RepeatExplorer (197.21×). The results show that this repeat is almost absent in the male genome (0.001%), while it covers more than 0.1% of the female genome suggesting that it is mainly localised on the W chromosome (Fig. 1). This assumption was confirmed by read mapping, which revealed even greater imbalance between the sexes, as coverage was 840× in females and only 2.5× in males. We also found a less frequent version of this element with a 213-bp long deletion. After mapping the reads to this “deleted” region, the coverage dropped to 698.5× in females, while it remained the same in males. The estimated copy number is 353 copies in females, out of which 60 have the deleted region, and 1–2 copies in males.

**Figure 3:**
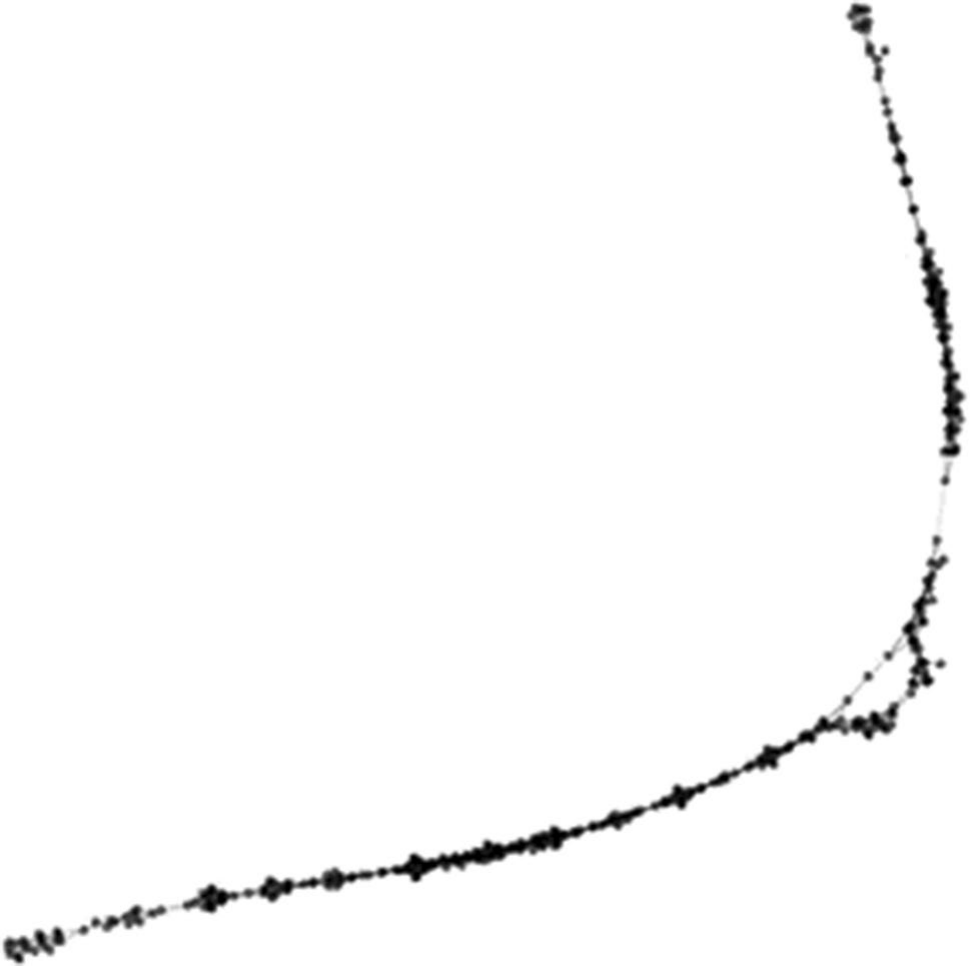
PRW Bel-Pao RepeatExplorer graph. Graph layout for cluster 79 generated by RepeatExplorer. Nodes represent individual illumina reads and edges their sequence overlap.

Both RepeatMasker and RepeatExplorer classified this cluster 79 repeat as an LTR transposon from the Bel-Pao group, based on the group-specific antigen (GAG), integrase (INT) and protease (PROT) domains. Although the graph of this repeat was linear (Fig. 3), indicating a less variable sequence of this repeat between copies, we could not recover all crucial protein-coding domains for this type of element, and the long terminal repeats were also missing.

NCBI BLASTN search yielded hits for *P.* *rhomboidaria* (98% identity) and for other Geometridae species, including the W chromosome of *Crocallis* *elinguaria* (73%) and the W chromosomes of two other Lepidoptera species, *Clostera* *curtula* (Notodontidae, 70%) and *Hydraecia* *micacea* (Noctuidae, 68%), with all sequences coming from the Tree of Life project^43^.

### PCR amplification of selected repeats from gDNA

**Figure 4:**
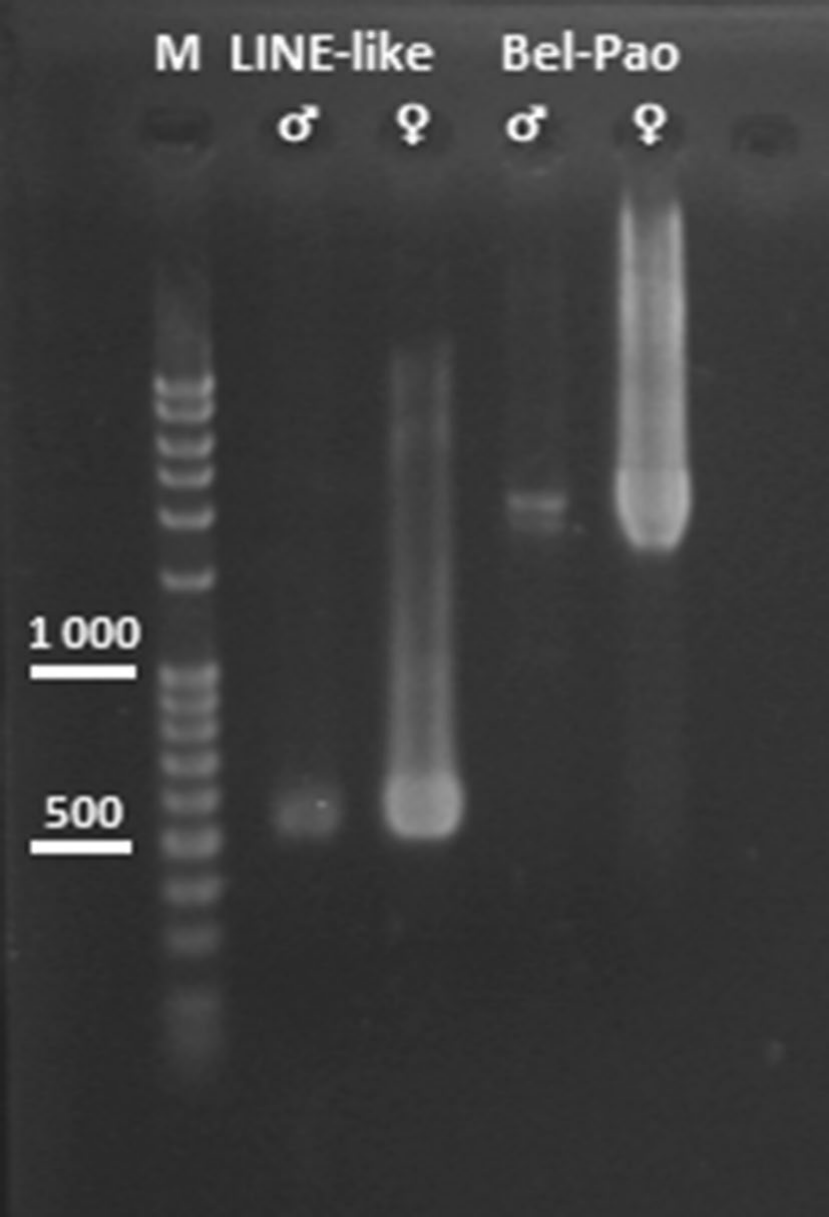
PCR amplification from genomic DNA of both sexes of *Peribatodes* *rhomboidaria*. Although the products are present in all samples, they are visibly stronger in females. Legend (from left): M—marker (100 bp DNA ladder, Invitrogen), PRW LINE-like male, PRW LINE-like female (540 bp), PRW Bel-Pao male, PRW Bel-Pao female (2118 bp; note a smaller band of 1905 bp carrying the deletion).

Both examined fragments were amplified by PCR (in the case of PRW Bel-Pao, we used the entire non-divided product from primer F1 to primer R2), using equal amounts of male and female gDNA. While the PCR products were present in both sexes, the female band was much stronger (Fig. 4). These results suggest that both elements are mainly present on the W chromosome.

### FISH mapping

**Figure 5:**
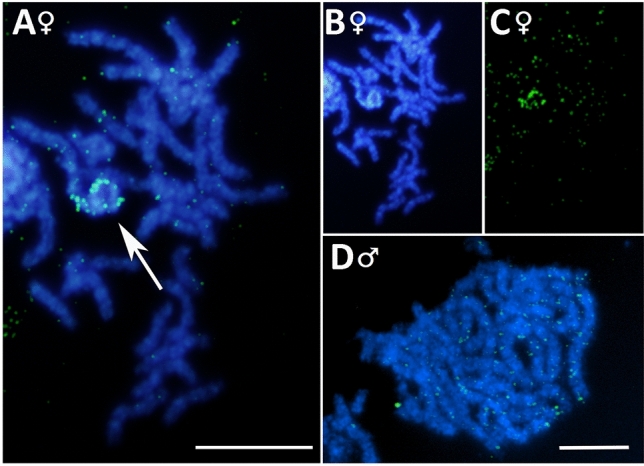
FISH mapping of PRW LINE-like on pachytene nuclei of *Peribatodes* *rhomboidaria*. (**A–C**) Female chromosomes with DAPI-positive W chromosome (arrow) accumulating hybridization signals and showing multiple scattered signals on other chromosomes, also seen in males (**D**). (**A,D**) Merged image of DAPI staining and probe (green), (**B**) DAPI staining, (**C**) probe (green). Bar = 10 μm.

**Figure 6:**
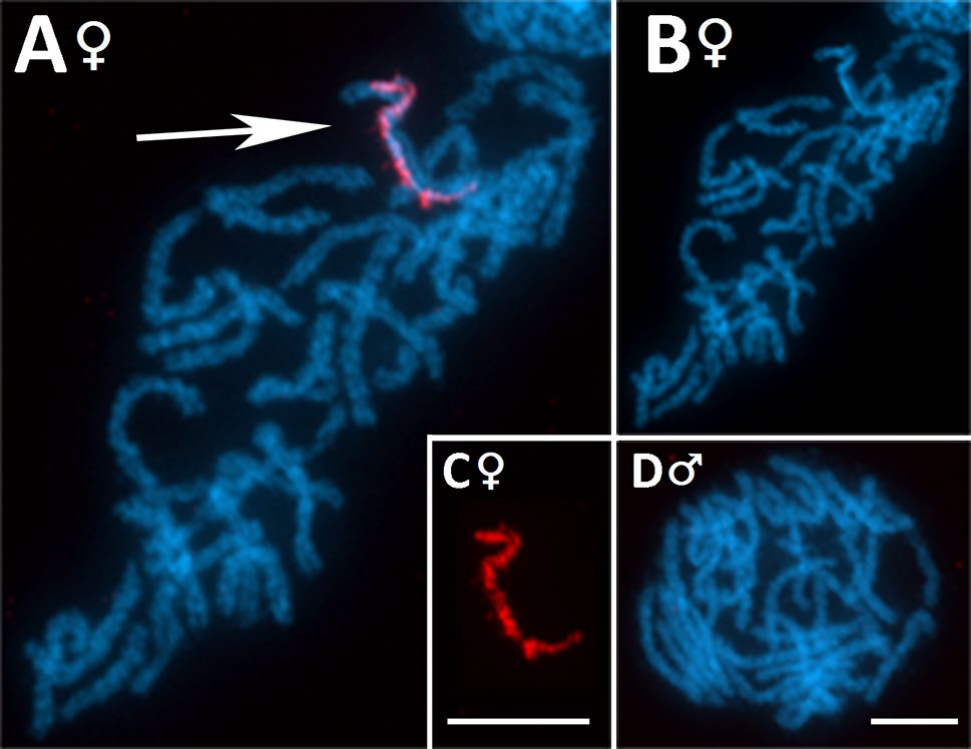
FISH mapping of PRW Bel-Pao on pachytene nuclei of *Peribatodes* *rhomboidaria*. (**A–C**) Female chromosomes with DAPI-positive W chromosome (arrow) showing strong hybridization signals, while no signals are visible on other chromosomes and on male pachytene chromosomes (**D**). (**A,D**) Merged image of DAPI staining and probe (red), (**B**) DAPI staining, (**C**) probe (red)—detail of the W chromosome. Bar = 10 μm.

To verify the predicted location of selected fragments, we carried out physical mapping using FISH. Indeed, both probes labelled the W chromosome (Figs. 5A, 6A), which was also identified by its heterochromatinization and corresponding DAPI positivity (Figs. 5B, 6B). In the case of PRW LINE-like (Fig. 5), the probe showed significant signal accumulation on the W chromosome, and multiple small scattered signals were also detected on other chromosomes, which were equally abundant in both sexes. In PRW Bel-Pao (Fig. 6), the probe hybridized exclusively on the W chromosome, which was labelled almost along its entire length, except for a small gap at one of the chromosomal ends. Accordingly, no signal was detected in males (Fig. 6D).

## Discussion

The genome size of *P.* *rhomboidaria* was estimated to 0.646 pg (corresponding to 631 Mb), which is similar to another geometrid species *Operophtera* *brumata* (645 Mb^44^) and falls within the range of known genome sizes in Lepidoptera from 273 Mb in *Danaus* *plexippus* (Nymphalidae) to 1.9 Gb in *Euchlaena* *irraria* (also Geometridae)^45^. However, the vast majority of species tend to have smaller genomes, similar to *B.* *mori* with 0.53 pg (corresponding to 518 Mb)^46^. The proportion of repetitive DNA in the genome of *P.* *rhomboidaria* was estimated to 44%; however, its actual volume is expected to be higher based on the genome size and repeat proportion in genomes of other Lepidoptera. For comparison, repeats in *D.* *plexippus* make up only 10.2% of the genome^47^, while in *B.* *mori* they comprise up to 46.8%^48^ and in *O.* *brumata* even more than half of the genome (53.5%)^44^. This discrepancy in *P.* *rhomboidaria* is likely caused by the RepeatExplorer analysis, which omits microsatellites and ancient diversified copies of mobile elements^49^. Although most of the repetitive sequences in *P.* *rhomboidaria* remain unclassified, similarly to genomes of other Lepidoptera the most abundant class of repeats are transposable elements. For instance, LINEs are particularly abundant in the two species of Geometridae, *P.* *rhomboidaria* (this study) and *O.* *brumata*^44^.

High occurrence of retrotransposons is a common feature of the W chromosome in Lepidoptera, although the number of species with available data is still limited^7,9,12^. In this study, we identified 10 putative W-enriched repeats of *P.* *rhomboidaria*, most of them being classified as LINE or LTR retrotransposons. Their identification was done by comparative analysis with the RepeatExplorer software, a novel approach for Lepidoptera as it only has been used for mapping satellite sequences in Crambidae^10^, and for the identification and characterization of W-enriched repetitive sequences in *Diatraea* *saccharalis* (Crambidae)^50^.

All these findings suggest that transposable elements are the main repeats with a tendency to colonize the W chromosomes of Lepidoptera and play the major role in their diversification. This phenomenon has already been observed in heterogametic sex chromosomes of many other organisms^51–53^. It seems that immediately after recombination around the sex-determining region is stopped, an accumulation of TEs follows^54,55^*.* This is one of the first events which may lead to the diversification of sex chromosomes (as seen e.g. in Ty3/Gypsy elements in papaya^56^), and even temporarily increase the size of the heterogametic chromosome at the early stage of differentiation^57^. The presence of TEs in the sex-determining region may also facilitate translocation of the sex-determining factor either via transposition or via ectopic recombination, potentially leading to the rise of a new sex chromosome pair^58,59^, or influence the expression of neighbouring genes. For instance, TE-induced methylation of a transcription factor realizes sex determination in melon^60^. Further, it is known that abundance of TEs is linked to the formation of heterochromatin, which may lead to the silencing of the whole chromosome (such as LINE in mammalian X chromosome inactivation^28^).

The expansion of TEs on the non-recombining sex chromosomes such as Y and W probably reflects the strongly reduced efficiency of natural selection on these chromosomes^61,62^. This is especially true for the W chromosomes of Lepidoptera, since meiotic recombination is completely absent in females^63–65^. Therefore, the abundant occurrence of TEs on the W chromosome in Lepidoptera is not surprising.

Moreover, the colonization of the W chromosomes of Lepidoptera by a particular type of retrotransposon is probably a rapid process with a random outcome, occurring independently even in closely related taxa^10^. However, it seems that at least for some retrotransposons, the W chromosome becomes the final destination, as they often genetically erode. This happens mainly through their recurrent uncoordinated insertions into already existing elements, which may destroy their functional domains. This, of course, also complicates their annotation.

PRW LINE-like element is a possible example of such an event. The complexity of the graph of the cluster 32 analysis with RepeatExplorer suggests a derived type of repeat with ambiguous annotation, as it contains two RT domains from different LINE elements and one DNA transposon Transib domain. Indeed, it is possible that there were originally several types of transposable elements that were repeatedly inserted into each other during their accumulation in the W chromosome (as in *B.* *mori*^66^), and this new ‘patchwork’ sequence could potentially amplify independently as a novel repeat (as suggested in legumes^67^).

Regarding the PRW LINE-like relative copy number differences between males and females, the RepeatExplorer analysis showed lower female enrichment than the coverage analysis in Geneious. These discrepancies are most likely due to the fact that RepeatExplorer analysed the entire cluster generated in silico, while only the cloned fragment (annotated as LINE CR1 by RepeatMasker) was used for Geneious read mapping. Since the RepeatExplorer graph may actually compile more types of monomers, it is possible that some of them are not accumulated on the W chromosome.

FISH mapping revealed a significant accumulation of the cloned PRW LINE-like sequence on the W chromosome, and its presence was also confirmed on other chromosomes, as many scattered signals were detected in both males and females. Accordingly, PCR results showed products in both sexes, although the female gDNA products were considerably stronger. These results are consistent with the fact that the PRW LINE-like female enrichment is less prominent in comparison to PRW Bel-Pao (Table 2), since it occurs profusely also on other chromosomes.

In the case of PRW Bel-Pao, it was classified as an LTR Bel-Pao element both by RepeatExplorer automatic annotation and RepeatMasker protein search. Both versions of this element are probably also non-functional and genetically eroded, as they lack the RT domain, long terminal repeats and in case of the less frequent deleted version also the PROT domain. Strong female enrichment of this transposon is very clear in both RepeatExplorer and Geneious coverage analyses. The difference between them is negligible and most likely technical, as the RepeatExplorer analysis is affected by a smaller sample size and a lower required percentage of identity between reads (90% vs. 95% in Geneious). Based on the coverage differences between the full-size fragment and the deleted region, we estimate that about 17% of the copies carry the deletion.

The PCR results strongly support the bioinformatic data. Two PCR products were found in both sexes, differing in volume and length. They correspond to a shorter, less abundant version carrying the deletion and a longer, more abundant full-length sequence. These results, combined with the coverage analysis data, therefore confirm that both deleted and non-deleted versions of this sequence are present on and off the W chromosome. Since it is unlikely that the original non-deleted version would mutate independently on different chromosomes, we hypothesize that the derived deleted version have migrated either to or from the W chromosome via an alternative transposition mechanism, such as ectopic recombination or with another mobile element.

As expected, the PCR products from the female gDNA were considerably stronger than those from the male gDNA. Accordingly, the FISH mapping revealed striking differences between the sexes and provided the final physical evidence for the accumulation of this repeat on the W chromosome. We did not observe hybridization signals on other chromosomes, but the probe highlighted almost the entire length of the W chromosome except for its terminal part, which did not contain heterochromatin. In our previous work, we showed results of comparative genomic hybridization (CGH), which revealed clear differences between the signals of the female gDNA probe in this terminal part and the heterochromatic part, proving that the latter part consists mainly of female-enriched sequences^30^. Thus, the PRW Bel-Pao repeat is probably one of the key components of the CGH results. Moreover, by colonizing the W chromosome, this repeat significantly contributes to its progressive differentiation.

To conclude, our data support previous findings that retrotransposons are the main cause of differentiation of the W chromosome in Lepidoptera. Moreover, we characterized two types of retrotransposons that accumulated on the W chromosome of *P.* *rhomboidaria* and we validated a novel approach to finding differences in repeat content using RepeatExplorer. Bioinformatic analysis corresponded very well with PCR and cytogenetic mapping, demonstrating the connectivity and mutual indispensability of these methods in providing a comprehensive insight. Despite the fact that Lepidoptera is a species-rich taxon, the W chromosome content is at least partly known in few species only. Therefore, the approach used in this work represents an alternative, elegant, and cost-effective tool for unveiling sequences which drive the W chromosome diversification.

## Supplementary Information

Supplementary Information.

## Data Availability

All examined sequences have been deposited into the GenBank with accession numbers provided in the Table 1 of “Materials and methods” section.
