# Supplementary material for: Accumulation of retrotransposons contributes to W chromosome differentiation in the willow beauty Peribatodes rhomboidaria (Lepidoptera: Geometridae)
Source: Sci Rep. 2023 Jan 11;13:534. doi: 10.1038/s41598-023-27757-3 (PMC9834309; doi:10.1038/s41598-023-27757-3)
Supplement: Supplementary file 1 — Supplementary Information. [file 41598_2023_27757_MOESM1_ESM.docx]

**Supplementary tables**

**Table S1. Primers used for PCR amplification of selected contigs.**

| **Primer name** | **Primer sequence** | **Product** |
| --- | --- | --- |
| *PRWline_F* | CGGAACACCTGAAAGAGCGA | PRW LINE-like (540 bp) |
| *PRWline_R* | GCATGTGTCCAGTCTCCTTG |  |
| *PRW_F1* | CGAATCCGTCCCCCTACTCT | PRW Bel-Pao part I (1394 bp) |
| *PRW_R1* | ATACAGCCACAAGTCCCACG |  |
| *PRW_F2* | GCAATTCTTCTTGCTCCCAAA | PRW Bel-Pao part II (1466 bp) |
| *PRW_R2* | GCTCCAGTATTTGAAGGCGG |  |

**Supplementary figures**

**
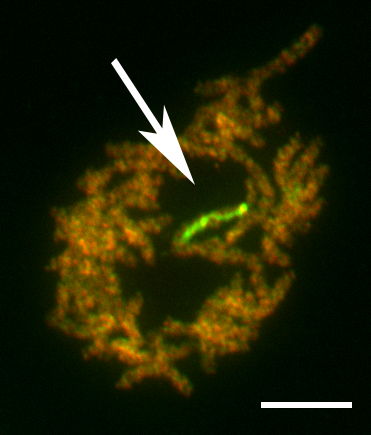
**

**Figure S1. Female pachytene nucleus of *Peribatodes rhomboidaria* after comparative genomic hybridization (CGH).** Male (red signals) and female (green signals) genomic DNAs were hybridized to female meiotic chromosomes. The W chromosome is strongly highlighted by the female probe, suggesting the presence of W-specific or highly W-enriched sequences. Arrow indicates the WZ bivalent. Bar = 10  μm.


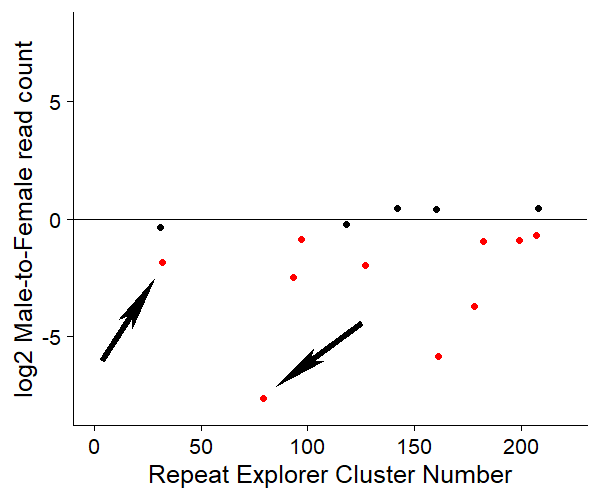


**Figure S2. RepeatExplorer results of comparative analysis.** Only clusters with statistically significant results between male and female read counts are shown (*t*-test, *P* < 0.05). Red dots represent clusters classified as putative W repeats. Clusters 32 and 79, which were further analysed are marked by arrows.
